# Supplementary material for: HIVprotI: an integrated web based platform for prediction and design of HIV proteins inhibitors
Source: J Cheminform. 2018 Mar 9;10:12. doi: 10.1186/s13321-018-0266-y (PMC5845081; doi:10.1186/s13321-018-0266-y)
Supplement: Supplementary file 2 — Additional file 2. Source code of HIVProtI web server. [file 13321_2018_266_MOESM2_ESM.zip › HIVprotI_Source-code/hivprotI/weka-3-6-6/doc/weka/associations/tertius/AttributeValueLiteral.html]

AttributeValueLiteral


---


|  |  |  |
| --- | --- | --- |
| **Overview** \| **Package** \| **Class** \| **Tree** \| **Deprecated** \| **Index** \| **Help** \| **Weka's home** | |  |
| PREV CLASS   **NEXT CLASS** | **FRAMES**    **NO FRAMES**     **All Classes** |  |
| SUMMARY: NESTED \| FIELD \| CONSTR \| METHOD | DETAIL: FIELD \| CONSTR \| METHOD |  |


---


## weka.associations.tertius Class AttributeValueLiteral

```
java.lang.Object
  weka.associations.tertius.Literal
      weka.associations.tertius.AttributeValueLiteral
```

**All Implemented Interfaces:**: java.io.Serializable, RevisionHandler

**Direct Known Subclasses:**: IndividualLiteral

---

``` public class AttributeValueLiteral extends Literal ```

**Version:**
:   $Revision: 1.6 $

**Author:**
:   Peter A. Flach, Nicolas Lachiche

**See Also:**: Serialized Form

---

**Field Summary**

**Fields inherited from class weka.associations.tertius.Literal**

`NEG, POS`


**Constructor Summary**

`AttributeValueLiteral(Predicate predicate,
java.lang.String value,
int index,
int sign,
int missing)`


| **Method Summary** | |
| --- | --- |
| `java.lang.String` | `getRevision()`             Returns the revision string. |
| `boolean` | `negationSatisfies(Instance instance)` |
| `boolean` | `satisfies(Instance instance)` |
| `java.lang.String` | `toString()` |

**Methods inherited from class weka.associations.tertius.Literal**

`getNegation, getPredicate, negative, positive, setNegation`

**Methods inherited from class java.lang.Object**

`equals, getClass, hashCode, notify, notifyAll, wait, wait, wait`

**Constructor Detail**

### AttributeValueLiteral

```
public AttributeValueLiteral(Predicate predicate,
                             java.lang.String value,
                             int index,
                             int sign,
                             int missing)
```


**Method Detail**

### satisfies

```
public boolean satisfies(Instance instance)
```

:   **Specified by:**: `satisfies` in class `Literal`

---


### negationSatisfies

```
public boolean negationSatisfies(Instance instance)
```

:   **Specified by:**: `negationSatisfies` in class `Literal`

---


### toString

```
public java.lang.String toString()
```

:   **Specified by:**: `toString` in class `Literal`

---


### getRevision

```
public java.lang.String getRevision()
```

:   Returns the revision string.

    :   **Returns:**: the revision


---


|  |  |  |
| --- | --- | --- |
| **Overview** \| **Package** \| **Class** \| **Tree** \| **Deprecated** \| **Index** \| **Help** \| **Weka's home** | |  |
| PREV CLASS   **NEXT CLASS** | **FRAMES**    **NO FRAMES**     **All Classes** |  |
| SUMMARY: NESTED \| FIELD \| CONSTR \| METHOD | DETAIL: FIELD \| CONSTR \| METHOD |  |


---
